# Variable Levels of Tolerance to Water Stress (Drought) and Associated Biochemical Markers in Tunisian Barley Landraces

**DOI:** 10.3390/molecules23030613

**Published:** 2018-03-08

**Authors:** Sameh Dbira, Mohamad Al Hassan, Pietro Gramazio, Ali Ferchichi, Oscar Vicente, Jaime Prohens, Monica Boscaiu

**Affiliations:** 1 Faculty of Mathematics, Physics and Natural Sciences of Tunis–El Manar University, Tunis 2092, Tunisia; dbira.sameh@gmail.com; 2 Institute of Plant Molecular and Cell Biology (IBMCP, UPV-CSIC), Universitat Politècnica de València, Camino de Vera s/n, 46022 Valencia, Spain; mohamad.alhassan@plantandfood.co.nz (M.A.H.); ovicente@ibmcp.upv.es (O.V.); 3 The New Zealand Institute for Plant & Food Research, Auckland 1025, New Zealand; 4 Institute for Conservation and Improvement of Valencian Agrodiversity, Universitat Politècnica de València, Camino de Vera s/n, 46022 Valencia, Spain; piegra@upv.es; 5 National Agronomy Institute-Tunis, 43 Avenue Charles Nicolle, Cité Mahrajène, Tunis 1082, Tunisia; ferchichi.ali1@yahoo.fr; 6 Mediterranean Agroforestry Institute, Universitat Politècnica de València, Camino de Vera s/n, 46022 Valencia, Spain; mobosnea@eaf.upv.es

**Keywords:** Ardhaoui landraces, biochemical markers, drought, genomic SSRs, *Hordeum vulgare*

## Abstract

Due to its high tolerance to abiotic stress, barley (*Hordeum vulgare*) is cultivated in many arid areas of the world. In the present study, we evaluate the tolerance to water stress (drought) in nine accessions of “Ardhaoui” barley landraces from different regions of Tunisia. The genetic diversity of the accessions is evaluated with six SSR markers. Seedlings from the nine accessions are subjected to water stress by completely stopping irrigation for three weeks. A high genetic diversity is detected among the nine accessions, with no relationships between genetic distance and geographical or ecogeographical zone. The analysis of growth parameters and biochemical markers in the water stress-treated plants in comparison to their respective controls indicated great variability among the studied accessions. Accession 2, from El May Island, displayed high tolerance to drought. Increased amounts of proline in water-stressed plants could not be correlated with a better response to drought, as the most tolerant accessions contained lower levels of this osmolyte. A good correlation was established between the reduction of growth and degradation of chlorophylls and increased levels of malondialdehyde and total phenolics. These biochemical markers may be useful for identifying drought tolerant materials in barley.

## 1. Introduction

Among cereals, barley (*Hordeum vulgare* L.) is outstanding for its tolerance to various stresses. Munns et al. [1] highlighted that barley is the most salt tolerant cereal, and its tolerance to drought or biotic stresses such as fungal disease is well-known [2]. There is a plethora of information on barley’s responses to abiotic stress, including recent studies and review articles on physiological, biochemical [2,3,4,5], metabolomics [6,7] and transcriptomics [8,9,10,11] aspects. Being an annual diploid plant species, with a small number of chromosomes (2 *n* = 14) and a huge range of genetic variability that accounts for different levels of stress tolerance, barley is a model species among crops for deciphering stress tolerance mechanisms, and hence has been subjected to many genetic studies in relation to stress tolerance, ranging from genetic variability analysis [12,13] to marker assisted selection in conjunction with the use of quantitative trait loci (QTLs) [14,15]. Because of its tolerance to drought compared to other major cereals, barley is an important crop in semi-arid regions for pasture and grain production, especially in the Middle East, North Africa, and southern and eastern Europe [16]. Barley is an important crop in Tunisia, where it is cultivated on 450,000 hectares in all of the country’s natural regions, amounting to 34–38% of the cereal cultivated area [17]. In southern Tunisia, where precipitation is very low, barley is the predominant cereal due to its tolerance to drought stress. In this region, there are many landraces, such as those included in the “Ardhaoui”, “Beldi”, “Djebali”, “Djerbi”, “Frigui”, “Sfira” or “Souihlis” landrace groups, which are well-known for their stress tolerance [18,19,20,21,22].

Due to desertification and soil salinization in many regions of the world, adapted materials with higher tolerance to abiotic stresses are needed [23]. Given that crop landraces are generally well adapted to the agroclimatic conditions of the region where they evolved and may contain a large genetic variation [24], they represent an interesting source for the selection of drought-tolerant materials. Barley landraces from Tunisia are genetically variable [18,25] and therefore are amenable to selection for drought tolerance, as long as diversity exists for this trait. In this respect, Ben Khaled et al. [18,26] found genetic variation for salinity tolerance among different landrace accessions of the “Ardhaoui” barley, which is autochthonous of southern Tunisia and is grown in areas with low precipitation.

In this work, we evaluate the tolerance to water stress (drought) of nine accessions of the “Ardhaoui” barley collected in different locations from four different regions of southern Tunisia with varying climatic conditions (rainfall, annual mean temperature, altitude). An analysis of genetic diversity with genomic SSRs was used to evaluate genetic diversity among the nine accessions. These materials were subjected to a controlled water stress tolerance treatment and compared to a control with the aim of identifying materials tolerant to drought and investigate the biochemical mechanisms involved in the tolerance to this stress. These results will be important for evaluating the variation for tolerance to drought, potential for selection and mechanisms of tolerance to drought of this resilient barley landrace.

## 2. Results

### 2.1. Molecular Characterization

**Figure 1:**
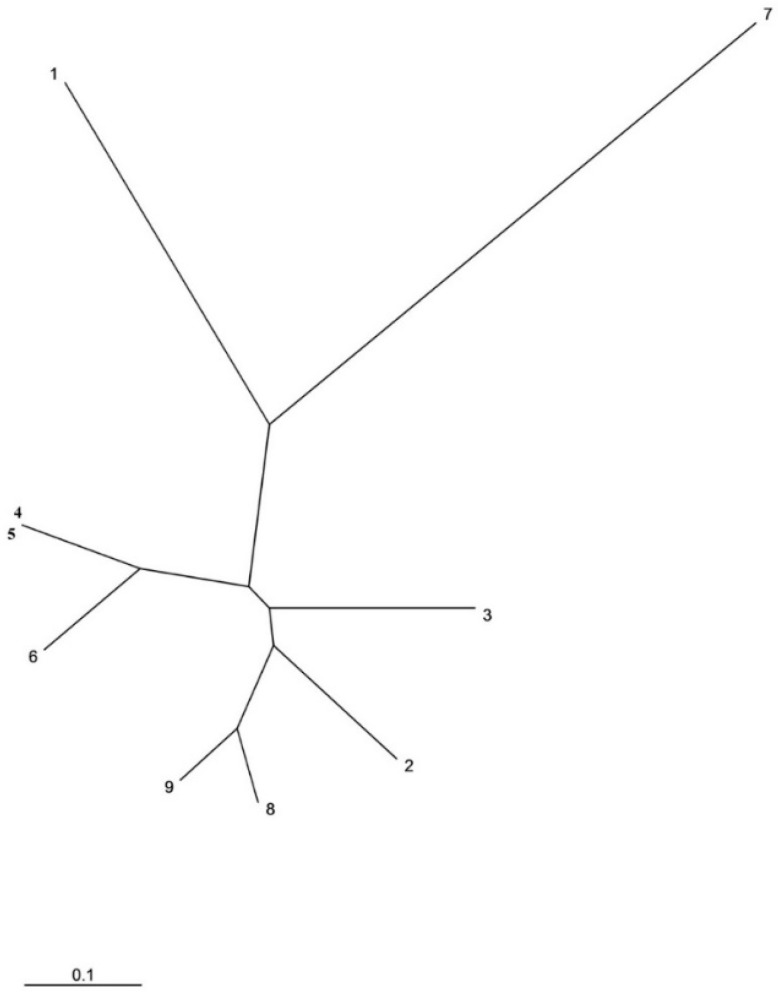
Unrooted neighbor-joining molecular phylogenetic tree of the nine accessions of the Ardhaoui barley landrace accessions under study based on six polymorphic genomic SSR markers [27]. Phenetic relationships were derived from genetic distances [28].

The six SSR loci were polymorphic in the set of nine accessions. The number of alleles (*N_a_*) ranged between 2 (GBM1256 and GBM1459) and 5 (GBM1461), and the frequency of the major allele was always ≥0.5 (Table 1). The number of effective alleles (*N_e_*) ranged between 1.52 (GBM1256) and 2.84 (GBM1221), while PIC values ranged between 0.286 (GBM1256) and 0.610 (GBM1461). The SSR genetic fingerprints revealed eight different genetic fingerprints among the nine accessions, resulting in eight branches in the phenogram of genetic relationships (Figure 1). Only two accessions (4 and 5) shared the same SSR profile.

The neighbor-joining phenogram displaying the genetic relationships among the nine accessions generally did not group accessions according to geographical distance or ecogeographical zone (Figure 1). For example, the two island accessions (1 and 2) cluster in different branches of the phenogram. In addition, accession 3 does not cluster together with the other genetically identical accessions situated in the same geographical area (4 and 5). In the same way, the two accessions from the littoral (6 and 7) are also situated in different branches of the dendrogram. Finally accessions 8 and 9, from the ridge zone, despite being located in the ridges at the northern and south extremes, respectively, of the Ksar mountains range cluster in the same branch of the dendrogram (Figure 1).

### 2.2. Growth Parameters

Significant differences were observed among accessions for the control plants for stem length, number of leaves, fresh weight, and dry weight (Table 2). Accessions 1 and 4 had the longest stems, with averages above 25 cm, while accessions 7 and 9 had the shortest stems with values below 22 cm. For the number of leaves, larger differences were observed, with accession 9 having over 30 leaves, while accession 1 just had eight leaves on average. Other accessions with a low number of leaves were accessions 8 and 2. Fresh weight was also very variable, with the largest values in accessions 3 (9.45 g) and 4 (8.69 g), and the lowest in accessions 1 (3.30 g) and 2 (4.46 g). Dry weight also displayed many differences among accessions, with highest values in accessions 3 (0.91 g) and 5 (0.85 g), and lowest in accessions 1 (0.22 g) and 8 (0.40 g) (Table 2).

Water stress induced a reduction in the stem length in all the barley accessions (Table 2), with a decrease ranging from 21% in accession 1 to 61% in accession 5. Accessions 2 and 9 also had a decrease lower than 30% for this trait, while accessions 3 and 4 had a reduction higher than 50%. When considering the absolute values for stem length for water-stressed plants the longest stems were observed in accessions 1 (20.56 cm) and 2 (17.55 cm), while the shortest in accessions 3 (9.71 cm) and 5 (9.74 cm).

The reduction in the number of leaves induced by water stress varied between 24% in accession 2 to 70% in accession 9 (Table 2). Fresh weight and dry weight decreased significantly in water-stressed plants in all accessions under study (Table 2). Accessions 3, 4, and 5 recorded the largest decrease for fresh weight, reaching over 80% in the three accessions, while accessions 1 and 2 experienced the smallest decrease in fresh weight percentage, with values below 50%. For dry weight, the largest reductions were observed in accessions 3 and 5, with reductions over 50% with respect to the control, while the lowest in accession 2, with a reduction of 17%. When looking at the absolute values of fresh weight under water stress, they ranged from 0.86 g in accession 5 to 2.58 g in accession 2. Accessions 6 and 1 ranked second and third in total fresh weight in stressed plants, with values of 1.84 g and 1.71 g, respectively.

Based on growth data, accession 2 can be considered as tolerant to drought, as this is the accession with lowest reductions in percentage in number of leaves, fresh weight and dry weight, and ranks second for stem length reduction (Table 2). In addition, under stress conditions it is the accession with largest number of leaves and greater fresh weight. Accessions 3, 4, 5, and 9 could be considered as susceptible to drought, as they had the highest reductions in percentage of fresh weight in stressed plants (over 90%) and also ranked high in the decreases in the percentage of number of leaves and stem length. Accessions 1, 6, 7 and 8 could be considered as intermediate in tolerance to water stress (Table 2). These differences were reflected in the phenotypes of the plants (i.e., leaf area and curling) subjected to water stress, which were mild in the tolerant landrace, severe in the susceptible ones, and moderate in the intermediate group ones.

### 2.3. Degradation of Photosynthetic Pigments

**Figure 2:**
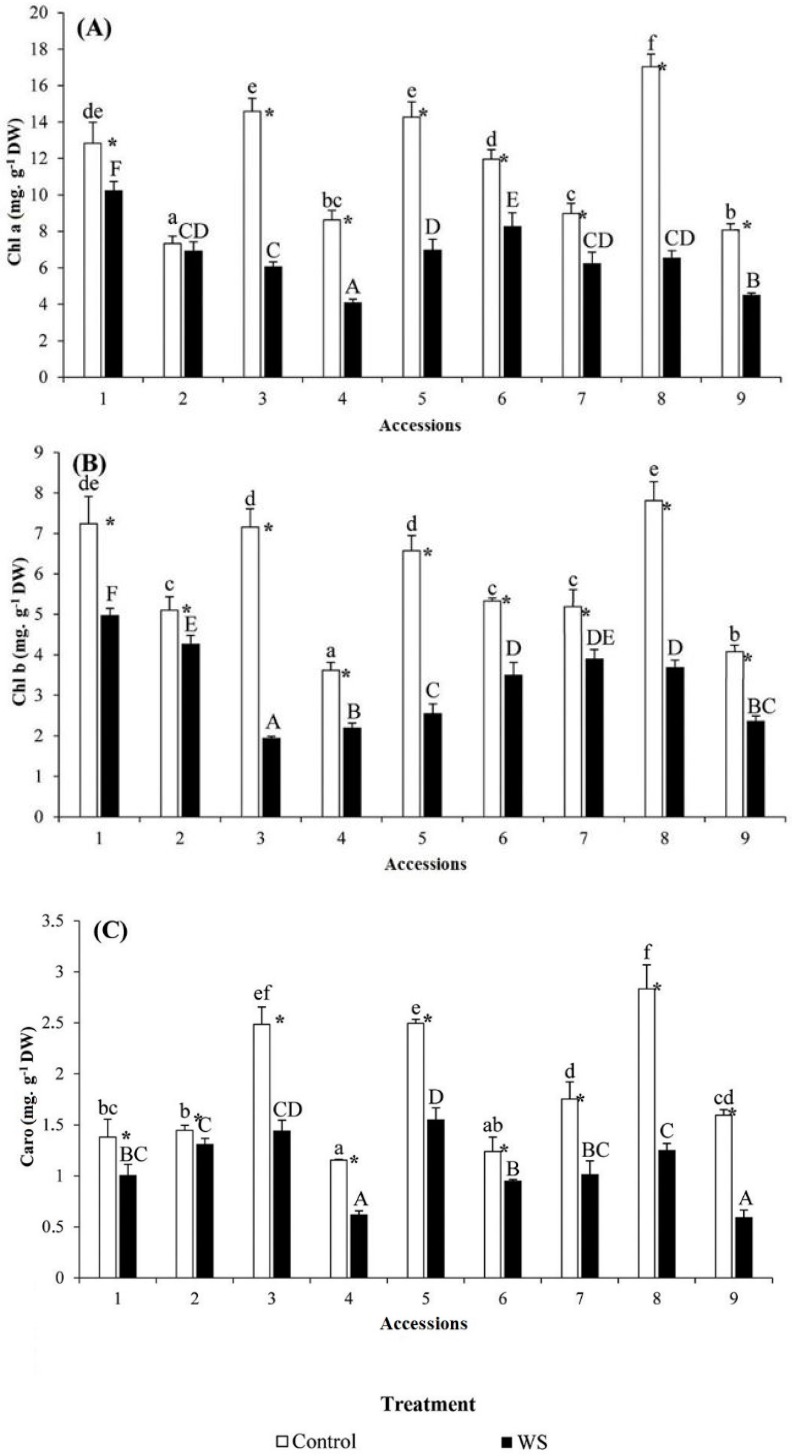
Variation in photosynthetic pigments content in the nine accessions of “Ardhaoui” barley landraces after three weeks of water stress (WS) treatment: (**A**) chlorophyll a (Chl a); (**B**) chlorophyll b (Chl b); and (**C**) total carotenoids (Caro). Error bars indicate SE (*n* = 5). For each accession, asterisks (*) indicate significant differences between control and WS treatments and different letters indicate significant differences among accessions undergoing the same treatment, according to the Tukey test (α = 0.05).

Differences were observed among accessions for the contents in Chl a, Chl b and Caro both under control and water stress conditions (Figure 2A–C, respectively). No apparent relationship was evident between tolerance to drought and content of chlorophylls or carotenoids either under control or stress conditions. Water stress induced a reduction in the levels of photosynthetic pigments Chl a, Chl b, and Caro in the leaves of all nine accessions (Figure 2A–C, respectively), but considerable differences were observed among the accessions in the level of the reduction. In this respect, reductions were the lowest for the three pigments in accession 2 (Figure 2A–C). Drought susceptible accessions 3, 4, 5, and 9, as well as intermediately susceptible accession 8 showed the biggest degradation in photosynthetic pigments under water stress (over 50% in some cases). Intermediately susceptible accessions 1, 6 and 7 generally had a lower reduction in pigments levels than the latter accessions.

### 2.4. Osmolytes and Antioxidant Contents

**Figure 3:**
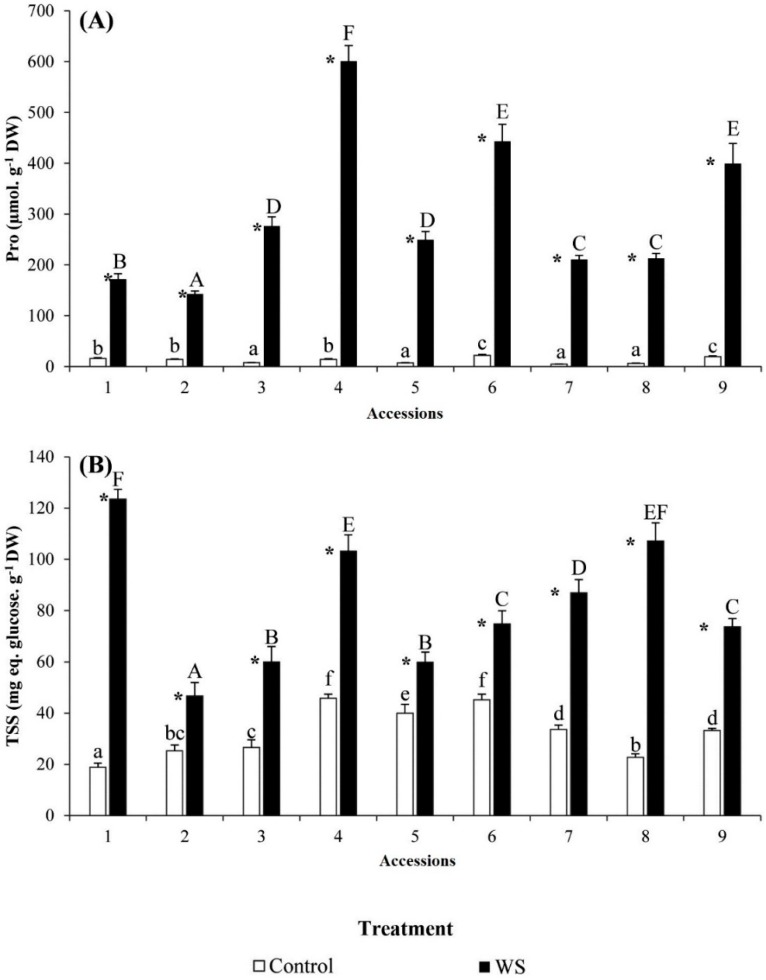
Variation in osmolytes concentrations in the nine accessions of “Ardhaoui” barley landraces after three weeks of water stress (WS) treatment: (**A**) proline (Pro); and (**B**) total soluble sugars (TSS). Error bars indicate SE (*n* = 5). For each accession, asterisks (*) indicate significant differences between control and WS treatments and different letters indicate significant differences among accessions undergoing the same treatment, according to the Tukey test (α = 0.05).

Significant differences were observed among accessions for proline (Pro) and total soluble sugars (TSS) both for control and water stress treatments (Figure 3A,B). No apparent association was found between levels of tolerance to drought and Pro or TSS contents in control plants. However, in water-stressed plants, the lowest accumulations of both osmolytes were observed in accession 2, with values significantly lower than those of the rest of accessions. Proline levels in water-stressed plants reached very high levels (nearly 150 to about 600 µmol g^−1^ DW), with relatively very high increases over the control (Figure 3A), indicating an osmotic role of Pro under stressful conditions. Considerable increases, although of lower magnitude than that found for Pro was observed for TSS. The highest values for Pro were detected in the susceptible accession 4, which had the biggest decrease in number of leaves, fresh weight, and water content in the leaves, while for TSS, the highest values were observed in intermediately susceptible accession 1 (Figure 3A,B).

**Figure 4:**
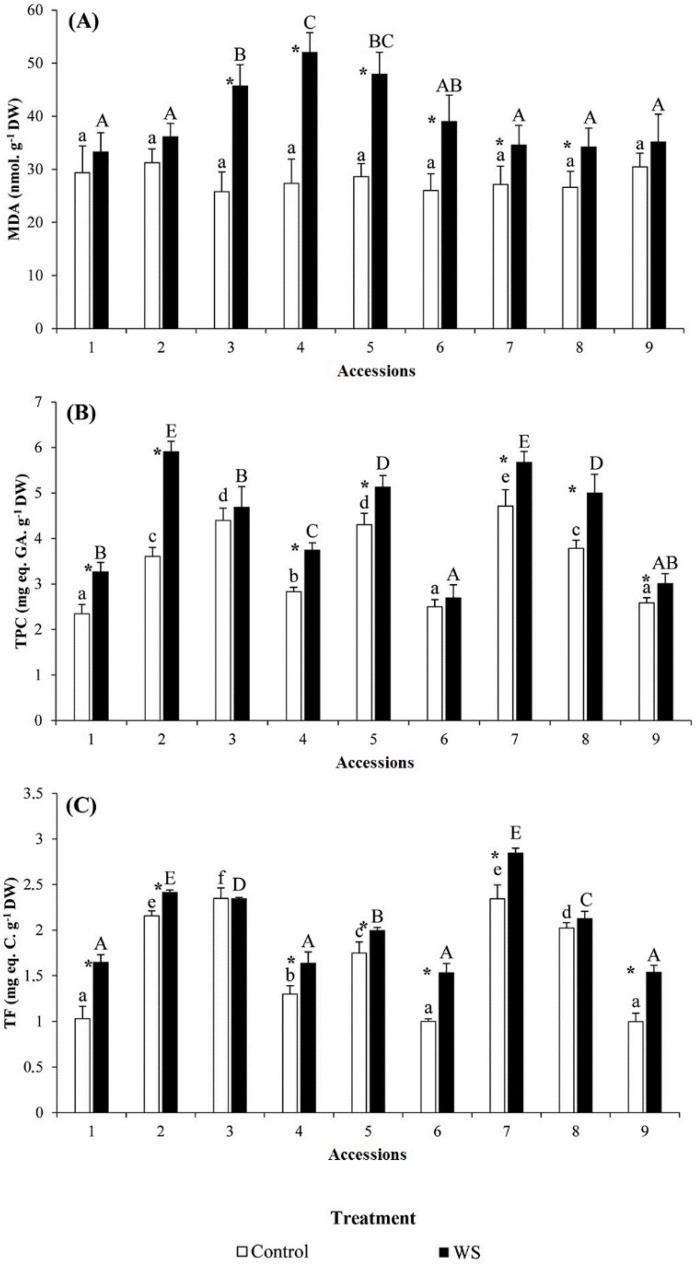
Variation in oxidative stress biomarker and chemical antioxidants concentrations in the nine Ardhaoui landrace accessions of barley after three weeks of water stress (WS) treatment: (**A**) malondialdehyde (MDA); (**B**) total phenolic compounds (TPC); and (**C**) total anti-oxidative flavonoids (TF). Error bars indicate SE. For each accession, asterisks (*) indicate significant differences between control and WS treatments and different letters indicate significant differences among accessions undergoing the same treatment, according to the Tukey test (α = 0.05).

Regarding the oxidative stress marker malondialdehyde (MDA), there were no significant differences among accessions in the control plants (Figure 4A). However, significant differences were observed among accessions in the drought stress treatment, with highest levels in susceptible accessions 4 and 5. In this respect, water-stressed plants increased the MDA levels moderately, although this difference was not significant for accessions 1, 2 and 9 (Figure 4A).

Significant differences were observed among accessions for total phenolic compounds (TPC) and total anti-oxidative flavonoids (TF) in control plants, although the levels observed were not associated to the levels of tolerance (Figure 4B,C). For water stressed plants, differences were also observed among accessions for these two antioxidants, which increased under water stress conditions (Figure 4B,C, respectively). Amazingly tolerant accession 2 had the highest levels of TPC, followed by intermediately tolerant accessions 7 and 8. Accession 2 also had the greatest increase in TPC levels due to water stress (Figure 4B). However, no apparent relationship exists between the levels of TF or their increase with tolerance to drought (Figure 4C), with highest absolute values in accession 7, followed by accession 2. The highest increases in TF were observed in accessions 1, 6 and 7.

## 3. Discussion

Drought is the most adverse abiotic factor affecting crops, and even the most optimistic scenarios forecast that in many regions of the world it will get worse in the future, due to the global warming [29]. Drought tolerance is a polygenic trait, affected by a complex array of genetic and environmental factors; as such, breeding for drought-tolerant crops is a rather difficult task [30]. Developing stress-tolerant cultivars while maintaining high productivity levels represents one of the main challenges for agricultural sciences [31]. Barley is one of the most stress-tolerant crops [2] with a large and diversified genetic pool [32], including several landraces adapted to arid and semi-arid environments [30]. Mediterranean barley landraces from arid and semi-arid regions represent valuable genetic resources that can be utilized in breeding programs for improving drought tolerance in elite cultivars [33]. Although barley is extensively cultivated in arid regions of Tunisia, the local landraces have been underutilized in drought tolerance research and as a valuable tool for improving stress tolerance through appropriate selection and breeding programs [34,35]. The local “Ardhaoui” barley, cultivated in southern Tunisia, is considered tolerant to drought [36] and salinity [18,23,35,37]; however variable responses to stress were detected among different landrace accessions indicating its genetic variability [18,26,37]. A preliminary study with three SSR markers revealed a high genetic diversity among 14 analyzed accessions of “Ardhaoui” [17]. In our work, we have confirmed that “Ardhaoui” barley is genetically very variable and that selection among different landrace accessions is feasible. With only six SSR markers we were able to distinguish eight different genetic profiles among nine accessions of the “Ardhaoui” barley, confirming the utility of these highly polymorphic markers for fingerprinting accessions from the same landrace group [27]. Although no SSR polymorphisms were found among two accessions (4 and 5), the fact that they present differences in growth parameters and biochemical traits is an indication that they are also genetically different.

Screening of reliable drought tolerance indicators is an important tool in the selection of tolerant genotypes [31]. Plant responses to drought are evident on multiple levels, including morphological, physiological and biochemical traits. The screening of these traits in genetically different materials may be a valuable tool in the selection of drought tolerant genotypes. Drought tolerance of a crop is often associated with its ability to maintain high yields in a water deficit environment. As yields rely on growth and development of plants, when selecting drought tolerant genotypes, the relative reduction of growth under stress is considered as an optimal indicator of adaptive capacity [38]. Unlike field trials, stress treatments applied during vegetative growth stage under greenhouse conditions are less expensive, easier to perform, and the severity of the stress applied may be easily controlled; as such are ideal for selection of efficient markers of stress in plants [38,39,40]. Inhibition of growth under stressful conditions is a general response of both stress-sensitive and stress-tolerant species, due to the diversion of resources from biomass accumulation towards the activation of stress defense mechanisms [41,42]. Drought, similar to other stresses, causes an imbalance in mineral nutrition, alteration of membrane permeability and cellular osmotic balance, and generation of oxidative stress [43].

Although all growth parameters analyzed in our study showed an inhibitory effect of water stress in all nine accessions, the most discriminant proved to be the total fresh weight (FW), whose reduction was almost double in the more sensitive accessions in comparison with the tolerant accession 2. Dry matter content of the aerial part (leaves and shoots) also significantly decreased in the water stress treatment in all accessions, although in a lower proportion than fresh weight, indicating a lower water content in the aerial tissues. In cereals, maintaining cell water potential is a main strategy of drought avoidance [30]. Altogether, growth parameters revealed that accession 2 is the most tolerant to drought, whereas accessions 3, 4, 5 and 9 are the most susceptible. Amazingly, tolerant accession 2 is not from the driest area, and the most susceptible accessions 3, 4, 5 and 9 were collected in the driest areas (average annual precipitation less than 200 mm). This fact suggests that other mechanisms for drought tolerance, such as fast growth and development that allow escaping the severe stress, may differ among these accessions [44].

Decreasing chlorophyll levels is considered as a symptom of oxidative stress and may be the result of pigment photo-oxidation and chlorophyll degradation [45]. Chlorophyll content represents one of the most frequently used tools for evaluating the severity of drought stress [46]. When comparing different genotypes, higher content of chlorophyll is generally associated with higher tolerance to stress [47,48]. In our case, chlorophyll degradation was observed in all accessions as a result of drought stress. However, tolerant accession 2, which had a smaller reduction of growth under drought, maintained higher levels of chlorophylls a, b and carotenoids under stress conditions.

Drought and salinity have an osmotic effect on plants due to the lowered soil water potential in the root zone which interferes with water absorption and maintenance of turgor. To counterbalance the high external osmotic pressure, plants synthesize and accumulate in their cytosol compatible solutes, commonly known as osmolytes [41]. Apart from their function in osmotic adjustment, osmolytes play other important roles such as low-molecular-weight chaperones, reactive oxygen species in (ROS) scavengers or signaling molecules [49,50]. Proline is one of the major osmolytes in many plant species, involved in responses to drought, salinity and other types of abiotic stress. Many studies indicated that proline is a main osmolyte in barley, starting with early studies by Singh et al. [51] and confirmed by metabolomics approaches [6]. When comparing different barley cultivars, higher levels of proline under stress in a particular genotype have often been linked with the ability to better withstand stress [37,52,53]. However, recent data point out that although playing an important role, proline accumulation in barley is a general conserved response not suitable as an indicator of better tolerance in this species [33,54]. The results we obtained agree with this statement, as the most tolerant accession had the lowest levels of proline under drought stress conditions.

Carbohydrates have also been reported as major osmolytes in barley [7], especially sucrose and malate [33,55] and some others such as ribose and 1-kestose [6]. We reported a strong increase in the levels of total soluble sugars, but no correlation was found with the relative stress tolerance of genotypes. On the contrary, the levels of MDA, which is considered as a suitable indicator of oxidative stress in barley [56] were much lower in the tolerant accession 2 than in the susceptible ones 3, 4 and 5. Regarding the antioxidants measured (total phenolics and total flavonoids), increases in both of them, particularly in total phenolics, in tolerant accession 2 compared to the susceptible accessions, suggests that increasing levels of these antioxidants could play a role in drought tolerance in this accession, as has been observed in wild barley [56].

In conclusion, our study reveals that wide variation exists for tolerance to drought in the Tunisian “Ardhaoui” barley and that highly tolerant genotypes can be selected that may be of interest for adaptation to climate change and yield stability under marginal conditions. In this respect, selection for drought tolerance among variable landrace accessions can be a rapid way to select adapted materials with increased drought tolerance. Our study also casts doubt on the utility of proline as indicator of drought tolerance in barley genotypes. Instead, it suggests that reduction of chlorophylls or MDA contents, or increases in total phenolics could be used as parameters for selecting drought-tolerant varieties of barley. The results obtained provide relevant information on the diversity and mechanisms of drought tolerance in the “Ardhaoui” barley, which is of interest for developing new barley materials with better tolerance to drought.

## 4. Materials and Methods

### 4.1. Plant Material

**Figure 5:**
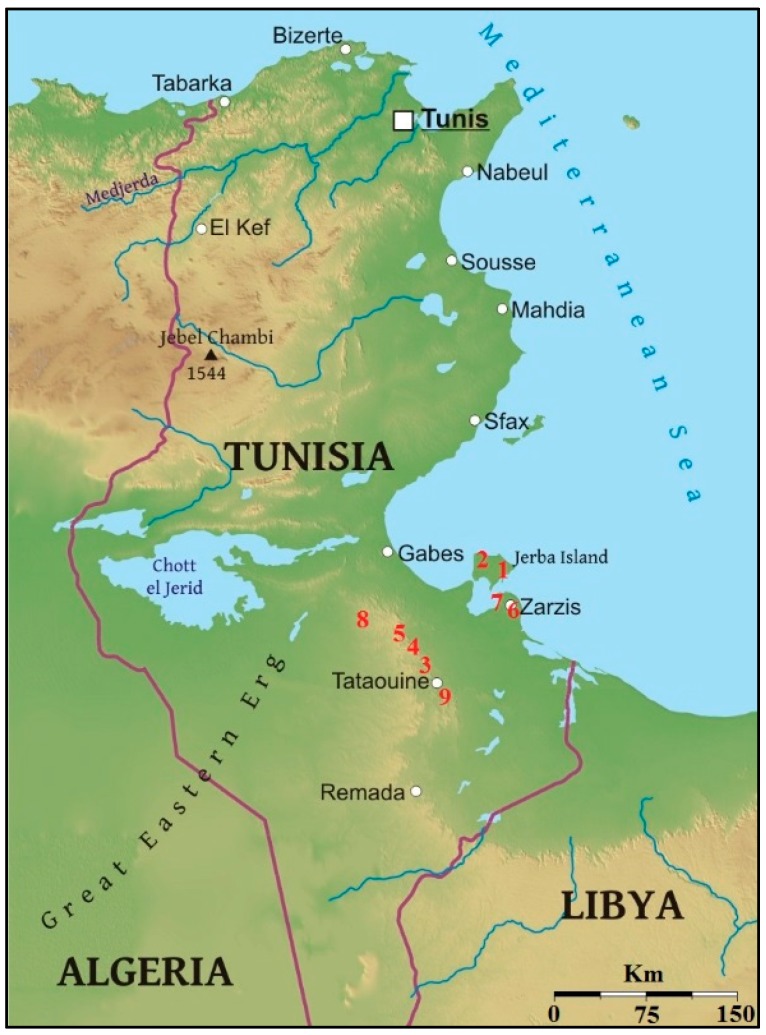
Location of the collection place of the nine accessions of “Ardhaoui” barley landraces evaluated.

Seeds of landrace accessions of the “Ardhaoui” barley from nine different locations in Southern Tunisia, which could be grouped into four different ecogeographical zones (Table 3), were used for the experiment. The nine zones have nearly identical mean annual temperature, but present different mean precipitations and altitudinal ranges. Accessions encoded as 1 and 2 come from the Island of Djerba on the Tunisian coast, while the rest come from mainland Tunisia. Accessions 3, 4, and 5, originate from the Ksar mountains, in Southern Tunisia, while accessions 6 and 7, came from the Southern Tunisian coast, close to the Island of Djerba. Populations 8 and 9, were collected from the ridges of the Ksar Mountains (Figure 5).

#### 4.1.1. Growth Conditions and Experimental Set-Up

Barley seeds were germinated, grown and treated in a greenhouse of the Institute of Plant Molecular and Cell Biology (IBMCP) at the Universitat Politècnica de València, Spain. A controlled growth environment was maintained, with regulated temperatures ranging between 17 and 23 °C, under a long-day photoperiod (16 h light/8 h dark); humidity ranged between 50% and 80%. The seeds were sown in seed trays containing a mixture of commercial peat and vermiculite (1:1). Immediately after emergence, young seedlings were transferred to individual polyethylene pots (Ø = 11 cm, with 1 L capacity) and were grown for three weeks. During this growth period, plants were watered with Hoagland’s nutrient solution [57]. Following the aforementioned growth period, water stress treatments were performed for three weeks on the nine accessions, with five replicas per treatment per accession. Control plants were watered twice a week with 0.25 L of nutrient solution per pot during the three weeks. The water stress treatment received no watering for the entire treatment period.

#### 4.1.2. DNA Extraction and Molecular Characterization

For each accession, genomic DNA was extracted from nearly 100 mg of young leaf tissue using the CTAB method [58]. DNA concentration was quantified, after electrophoresis on a 1.0% agarose gel, using a Nanodrop ND-1000 (Nanodrop Technologies, Wilminton, DE, USA) spectrophotometer. Samples were adjusted to a DNA concentration of 20 ng/µL. The quality of DNA was evaluated through the 260/280 nm and 260/230 nm absorbance ratios [59]. Six genomic highly polymorphic SSR markers developed by Stein et al. [27], which map in different linkage groups in the barley genetic map, were used to screen the nine barley accessions under study. An M13-tailed forward primer was used in combination with a standard M13 primer dye-labeled with FAM, NED, or VIC fluorophores at its 5′-end. PCR amplifications were performed in a total volume of 12 µL with 20 ng of DNA, 1.5 mM MgCl_2_, 0.05 µM of forward primer, 0.25 µM of reverse primer, 0.2 µM of fluorescent M-13 primer, 0.2 mM dNTPs, and 0.20 µL Taq DNA polymerase (TaKaRa Bio Inc., Kyoto, Japan), at a concentration of 5 U/µL. Amplifications were carried out in an Eppendorf Mastercycler ep gradient S (Eppendorf AG, Hamburg, Germany) thermocycler. Amplification procedure via the thermocycler consisted of an initial step at 94 °C for 5 min; 35 cycles of 94 °C for 30 s, 30 s at 62 °C, and 72 °C for 45 s; and final 10 min extension at 72 °C.

PCR products were diluted in formamide and analyzed on an automated DNA sequencer ABI PRISM 3100-Avant with a GeneScan 600LIZ (Applied Biosystems, Foster City, CA, USA) size standard. The data were analyzed using the GeneScan software (Applied Biosystems) to obtain the electropherograms and polymorphisms were analyzed with Genotyper DNA Fragment Analysis software (Applied Biosystems).

#### 4.1.3. Measuring of Growth Parameters

Stem length was measured every three days after the initiation of treatments. In addition, the number of leaves was monitored throughout the treatments period. At the end of the treatments (three weeks), all plants were harvested, and the vegetative parts were weighed individually on a precision balance. Part of the fresh material was weighed (fresh weight, FW) before being dried at 65 °C, until it reached constant weight and then weighed again (dry weight, DW). The dry weight percentage (DW; %) was calculated as (DW/FW) × 100.

#### 4.1.4. Photosynthetic Pigments and Biochemical Parameters Measurement

Chlorophyll a (Chl a), chlorophyll b (Chl b), and total carotenoids (Caro), were quantified following the protocol described by Lichtenthaler and Welburn [60] using 100 mg of fresh leaf material. The concentrations of Chl a, Chlb and Caro were calculated using Lichtenthaler and Welburn [60] equations. Final values were expressed in mg g^−1^ DW. Proline (Pro), total soluble sugars (TSS), malondialdehyde (MDA), total phenolics content (TPC), and total flavonoids (TF) contents were determined in dry leaf material. Pro was measured according to the method described by Bates et al. [61] using glacial acetic acid and ninhydrin. Pro concentration was expressed as µmol g^−1^ DW. TSS was measured following the method devised by Dubois et al. [62] using dried material. TSS contents were expressed as “mg equivalent of glucose” per gram of DW. MDA, a final product of membrane lipid peroxidation and a reliable marker of oxidative stress [63], was determined following the protocol designed by Hodges et al. [64]. MDA concentration was calculated using the equations described in Hodges et al. [64]. TPC was measured following Blainski et al. [65] using the Folin-Ciocalteu reagent. TPC was expressed in equivalents of gallic acid, which was used as standard (mg. eq GA g^−1^ DW). TF was quantified following the method described by Zhishen et al. [66]. The amount of total antioxidant flavonoids was expressed in equivalents of catechin, which was used for the standard curve (mg eq C g^−1^ DW).

### 4.2. Data Analysis

For genetic characterization of the nine accessions of “Ardhaoui” barley landraces, the number of polymorphic alleles (*N_a_*), frequency of the predominant allele (*f*), effective number of alleles (*N_e_*), and polymorphic information content (*PIC*) was determined for each SSR locus using the PowerMaker software [67]. Genetic similarities were calculated and a neighbor-joining phenogram was built using genetic distances with the PowerMaker software [67] and plotted using TreeView software [68].

Morphological and biochemical data were analyzed using the program Statgraphics Centurion v.16 (Statgraphics Technologies, Inc., The Plains, VA, USA). The significance of the differences among treatments was tested by ANOVA tests, and post hoc comparisons were made using the Tukey HSD test at *p* < 0.05. The same kind of analysis was performed between plants from different accessions undergoing the same treatment (control or water stress).

## Figures and Tables

**Table 1:** Diversity statistics for the six genomic SSR markers [27] and genetic profiles (allele sizes) based on for the nine “Ardhaoui” barley accessions evaluated in the present work.

Diversity Statistics	SSR Marker					
	GBM1176	GBM1221	GBM1256	GBM1405	GBM1459	GBM1461
Linkage group	5H	4H	6H	3H	2H	1H
Number of alleles (*N_a_*)	4	4	2	3	2	5
Frequency of the major allele (*f*)	0.667	0.500	0.778	0.667	0.500	0.556
Effective number of alleles (*N_e_*)	2.05	2.84	1.53	1.98	2.00	2.79
Polymorphic information content (*PIC*)	0.473	0.593	0.286	0.438	0.375	0.610

**Table 2:** Variation (average ± SE) in growth parameters in the nine accessions of the Ardhaoui barley landrace in the control and water stress (WS) treatments after three weeks of initiation of the water stress treatment.

Accession	Treatment	Stem Length (cm) ^a^	Number of Leaves ^a^	Fresh Weight (g) ^a^	Dry Weight (g) ^a^
1	Control	25.90 ± 1.29	8.00 ± 0.95	3.30 ± 0.41	0.22 ± 0.02
	WS	20.56 ± 2.15 *	5.26 ± 0.41 *	0.71 ± 0.11 *	0.17 ± 0.01 *
2	Control	24.15 ± 1.12	13.00 ± 1.15	4.46 ± 0.70	0.53 ± 0.05
	WS	17.55 ± 0.45 *	9.90 ± 0.69 *	2.58 ± 0.22 *	0.44 ± 0.03 *
3	Control	23.15 ± 1.84	22.25 ± 0.76	9.45 ± 0.51	0.91 ± 0.02
	WS	9.71 ± 0.79 *	9.54 ± 0.33 *	1.22 ± 0.07 *	0.37 ± 0.01 *
4	Control	25.90 ± 3.30	23.40 ± 0.92	8.69 ± 0.71	0.82 ± 0.05
	WS	11.21 ± 0.34 *	8.53 ± 0.55 *	0.86 ± 0.04 *	0.48 ± 0.02 *
5	Control	24.89 ± 0.78	18.00 ± 0.95	7.98 ± 0.29	0.85 ± 0.03
	WS	9.74 ± 0.96 *	9.86 ± 0.88 *	1.26 ± 0.07 *	0.40 ± 0.03 *
6	Control	22.08 ± 0.64	19.60 ± 0.81	6.87 ± 0.60	0.50 ± 0.04
	WS	13.00 ± 0.19 *	8.77 ± 0.59 *	1.84 ± 0.22 *	0.32 ± 0.02 *
7	Control	21.63 ± 1.01	17.80 ± 0.37	7.36 ± 0.67	0.63 ± 0.05
	WS	12.11 ± 1.65 *	8.50 ± 0.64 *	1.57 ± 0.19 *	0.33 ± 0.02 *
8	Control	22.02 ± 1.39	11.66 ± 0.68	5.71 ± 0.38	0.40 ± 0.02
	WS	15.50 ± 1.03 *	6.16 ± 0.35 *	1.37 ± 0.24 *	0.30 ± 0.03 *
9	Control	19.88 ± 0.56	30.20 ± 2.13	6.96 ± 0.47	0.61 ± 0.03
	WS	15.20 ± 0.42 *	9.31 ± 0.34 *	1.37 ± 0.19 *	0.44 ± 0.04 *

^a^ An asterisk (*) indicates significant differences within accession between control and WS treatments, according to the Tukey test (α = 0.05).

**Table 3:** Altitude, annual mean temperature (T) and annual rainfall (R) of the accessions of “Ardhaoui” barley landraces under study (The World Bank Group, Climate Change Knowledge Portal, reference period 1990–2012).

Code	Accession	Zone	Alt. (m)	T (°C)	R (mm)
1	Midon	Island	50	21.25	311.25
2	El May	Island	35	20.37	336.13
3	Guermassa	Mountain	349	21.12	151.50
4	Ksar Hdada	Mountain	328	20.46	181.91
5	Graguer	Mountain	255	21.01	161.45
6	Zarzis	Coast	11	20.86	259.57
7	Eljdaria	Coast	14	20.47	204.05
8	Ksar Bayada	Ridge	185	21.08	239.14
9	Smar Tataouine	Ridge	85	21.09	158.20
